# Tuning the immune response: sulfated archaeal glycolipid archaeosomes as an effective vaccine adjuvant for induction of humoral and cell-mediated immunity towards the SARS-CoV-2 Omicron variant of concern

**DOI:** 10.3389/fimmu.2023.1182556

**Published:** 2023-04-14

**Authors:** Tyler M. Renner, Bassel Akache, Matthew Stuible, Nazanin Rohani, Yuneivy Cepero-Donates, Lise Deschatelets, Renu Dudani, Blair A. Harrison, Jason Baardsnes, Izel Koyuturk, Jennifer J. Hill, Usha D. Hemraz, Sophie Régnier, Anne E. G. Lenferink, Yves Durocher, Michael J. McCluskie

**Affiliations:** ^1^ National Research Council Canada, Human Health Therapeutics, Ottawa, ON, Canada; ^2^ National Research Council Canada, Human Health Therapeutics, Montreal, QC, Canada; ^3^ National Research Council Canada, Aquatic and Crop Resource Development, Montreal, QC, Canada

**Keywords:** sulfated archaeal glycolipid (SLA), archaeosome, adjuvant, SARS-CoV-2, spike subunit vaccine, omicron, AddaS03, variants of concern (VOC)

## Abstract

Liposomes composed of sulfated lactosyl archaeol (SLA) have been shown to be a safe and effective vaccine adjuvant with a multitude of antigens in preclinical studies. In particular, SLA-adjuvanted SARS-CoV-2 subunit vaccines based on trimeric spike protein antigens were shown to be immunogenic and efficacious in mice and hamsters. With the continued emergence of SARS-CoV-2 variants, we sought to evaluate next-generation vaccine formulations with an updated antigenic identity. This was of particular interest for the widespread Omicron variant, given the abundance of mutations and structural changes observed within its spike protein compared to other variants. An updated version of our resistin-trimerized SmT1 corresponding to the B.1.1.529 variant was successfully generated in our Chinese Hamster Ovary (CHO) cell-based antigen production platform and characterized, revealing some differences in protein profile and ACE2 binding affinity as compared to reference strain-based SmT1. We next evaluated this Omicron-based spike antigen for its immunogenicity and ability to generate robust antigen-specific immune responses when paired with SLA liposomes or AddaS03 (a mimetic of the AS03 oil-in-water emulsion adjuvant system found in commercialized SARS-CoV-2 protein vaccines). Immunization of mice with vaccine formulations containing this updated antigen with either adjuvant stimulated neutralizing antibody responses favouring Omicron over the reference strain. Cell-mediated responses, which play an important role in the neutralization of intracellular infections, were induced to a much higher degree with the SLA adjuvant relative to the AddaS03-adjuvanted formulations. As such, updated vaccines that are better capable of targeting towards SARS-CoV-2 variants can be generated through an optimized combination of antigen and adjuvant components.

## 1 Introduction

The ongoing COVID-19 pandemic continues to put pressure on the global community, with continued strain on health care systems worldwide. Despite the approval and widespread uptake of a variety of vaccines, morbidity and mortality related to SARS-CoV-2 infections continue at a high rate. Several factors contribute to this, such as the emergence of novel viral variants and the rapid waning of immunity in the wider population, whether generated through vaccination or environmental exposure to the virus. Kinetic analyses have illustrated that immunological responses towards SARS-CoV-2 spike diminish considerably within months post-vaccination (1, 2). Furthermore, the evolution of variants has selected for viral strains capable of circumventing previously developed neutralizing antibodies, with Omicron and its many subvariants continuing to show high infectivity rates in vaccinated populations (1, 3). This highlights the need for continued development of next generation vaccines capable of enhancing immunological protection to these highly evolved SARS-CoV-2 strains.

While mRNA and adenoviral-based SARS-CoV-2 vaccine products were developed rapidly and have been widely employed, they have been associated with rare but severe side effects (4–6). Protein-based vaccines have a proven track record of safety and efficacy with a number approved for influenza (e.g. Pandemrix and Arepanrix (7)) and more recently COVID-19, including Nuvaxovid (8), VidPrevtyn Beta and Covifenz (9, 10). These COVID-19 vaccines utilize a reference strain-based spike antigen and, as is typical with protein vaccine formulations, also include immunostimulatory adjuvants to enhance the immune responses. Nuvaxovid uses the saponin-based Matrix-M as an adjuvant, while VidPrevtyn Beta and Covifenz are adjuvanted with a squalene-based oil-in-water emulsion AS03. The COVID-19 pandemic has revealed the limited availability of effective commercialized adjuvants, highlighting the need to develop new manufacturable adjuvants with strong activity profiles. Archaeosomes are a class of experimental adjuvants based on liposomes composed of archaeal lipids, which uniquely contain the archaea-specific ether-linked isoprenoid phytanyl chains. These lipids have inherent immune-stimulating properties not seen with conventional liposomes composed of standard ester lipids (11). Sulfated lactosyl archaeol derived archaeosomes (SLA) are composed of a semi-synthetic glycolipid containing the archaeol core linked to a lactose sugar with the sulfate polar head group. They demonstrate a favourable preclinical safety profile, while inducing robust humoral and cellular antigen-specific immune responses to various antigens including those based on SARS-CoV-2 spike (12–14).

Our group has previously demonstrated production by CHO cells of high yields of a resistin-trimerized SARS-CoV-2 spike vaccine antigen, referred to as SmT1, which is customizable to multiple variants (14, 15). Using this platform, we demonstrated that SLA-adjuvanted vaccines generate robust humoral and cellular antigen-specific immune responses that were protective in a hamster SARS-CoV-2 virus challenge model (14). Upgrading the vaccine formulations with SARS-CoV-2 Beta or Delta SmT1 antigens further improved the neutralization responses elicited against the corresponding variant of concern (VOC) (16). For the more recently emerged Omicron variant, it remained to be determined if the significant sequence divergence from previous variants would impact the immunogenicity of the corresponding spike antigen or our ability to produce it efficiently using our resistin-trimerized spike antigen production platform. Herein, we evaluated the immunogenicity of Omicron SmT1 adjuvanted with SLA in a preclinical mouse model and compared the elicited responses to those obtained with similar formulations adjuvanted with AddaS03, an AS03 mimetic.

## 2 Results

**Figure 1:**
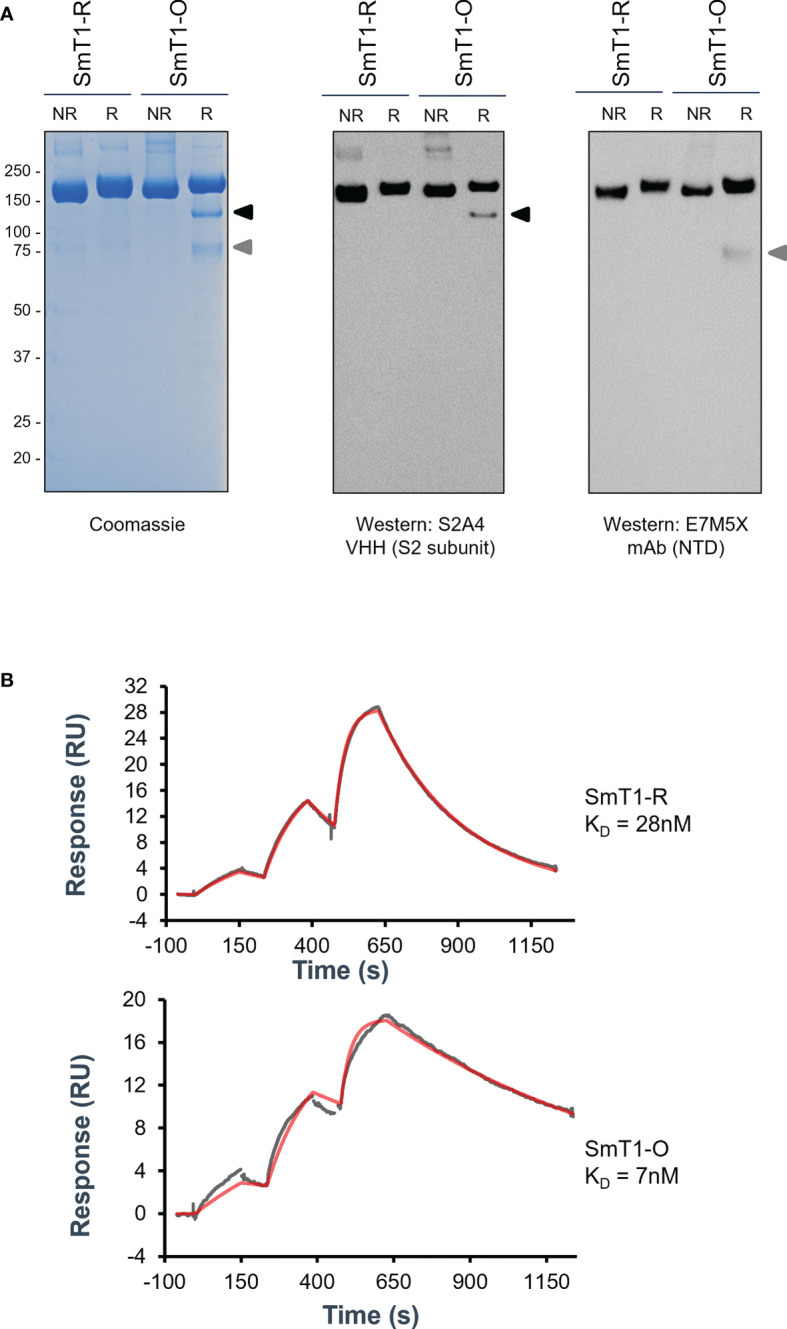
Purified SmT1-O (Omicron) spike protein exhibits non-full-length fragments by reducing SDS-PAGE and increased affinity for ACE2 by SPR compared to reference strain SmT1-R. SDS-PAGE of 3 µg total protein analyzed *via* **(A)** Coomassie stain (Left panel) or Western blot targeting either the S2 subunit (Middle panel) or N-terminal domain (Right panel) domains. R = Reducing; NR = Non-Reducing. **(B)** Surface plasmon resonance binding sensorgrams showing the 1:1 binding simulated best fit (red) and experimental data (gray) fit for the SmT1-R (top) and SmT1-O (bottom) interaction with human ACE2.

Using our CHO^55E1^ transient gene expression platform, we generated a version of SmT1 based on the Omicron variant (B.1.1.529 or BA.1) spike sequence, referred to hereafter as SmT1-O, to compare to the corresponding reference strain spike construct, SmT1-R (15). Changes to the SmT1 sequence did not significantly alter productivity, as SmT1-O protein was produced at a similar yield (55 mg/L in supernatant before purification) to that observed previously for other variants of SmT1. By sodium dodecyl sulfate–polyacrylamide gel electrophoresis (SDS-PAGE), the SmT1-R protein was observed, as expected, as a predominant full-length band of 180-200 kDa under reducing or non-reducing conditions. A full-length band of similar molecular weight is also observed for SmT1-O, but under reducing conditions, two smaller fragments were also present. Notably, these smaller bands were not seen in the absence of reducing agent, indicating that these fragments likely remain associated *via* disulfide bonds and together have a molecular weight similar to full-length SmT1-R (**Figure 1A**). Western blotting with antibodies specific for the spike N-terminal domain (NTD; mAb E7M5X) and S2 subunit (V_H_H S2A4) confirmed that the smaller bands are fragments of the spike protein, with each fragment only recognized by one of the two antibodies. To confirm the spike protein’s binding profile to ACE2, we measured affinity for human ACE2 by surface plasmon resonance (SPR): the dissociation constant (K_D_) for SmT1-O binding to ACE2 is 7 nM compared to 28 nM for SmT1-R, a result of a slower off-rate for SmT1-O. This indicates that SmT1-O structure is still recognizable by ACE2 despite the presence of partially cleaved products (**Figure 1B**). The higher affinity of SmT1-O *vs*. SmT1-R is consistent with data from other groups showing increased affinity of Omicron spike for ACE2, which is very likely the result of the numerous mutations present in the Omicron spike RBD sequence (17, 18).

To assess the immunogenicity of this protein, mice (n = 10 per group) were immunized at days 0 and 21 with vaccine formulations containing 1 or 3 µg of SmT1-O adjuvanted with SLA or AddaS03. An additional group received 1 µg of SmT1-R (reference strain) + SLA. This would serve as a comparative bridge to our previous studies (14, 16) and allow us to compare the neutralization activity induced by SmT1-O *vs*. SmT1-R in a head-to-head setting.

### 2.1 Cell-mediated immunity

**Figure 2:**
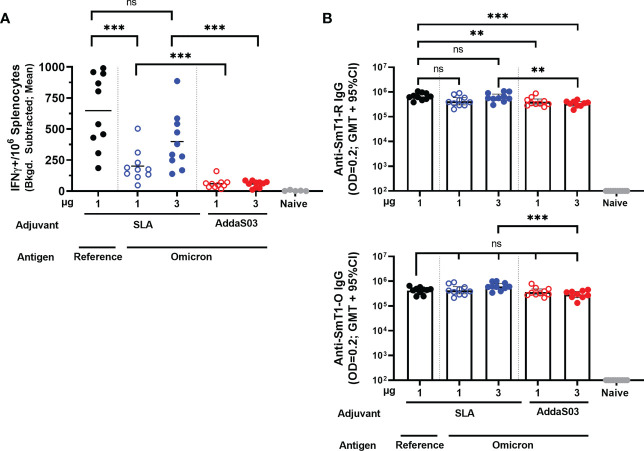
Adjuvanted formulations of SmT1-O and SmT1-R elicit cellular and humoral antigen-specific immune responses. C57BL/6 mice (n = 10/group) were immunized i.m. with SmT1-R or SmT1-O adjuvanted with SLA or AddaS03 on days 0 and 21. **(A)** Splenocytes were harvested on Day 28 and analyzed by IFN-γ ELISpot when stimulated by spike peptide pools. Values obtained with media alone were subtracted from those measured in the presence of the peptides. Grouped data are presented as a mean. **(B)** Serum was also collected on Day 28 and analyzed by ELISA against SmT1-R (top graph) and SmT1-O (bottom graph) to determine the antibody titers on Day 28. Grouped data are presented as geometric mean + 95% confidence interval. In all graphs, statistical significance of differences is shown as: ns = not significant, **p < 0.01 and ***p < 0.001 by one-way ANOVA followed by Tukey’s multiple comparisons test when compared to the other adjuvant with an equivalent dose of SmT1-O or the SmT1-R containing vaccine.

To determine the level of antigen-specific cellular immunity induced by these vaccines, an IFN-γ ELISpot was performed on splenocytes collected on Day 28. A peptide pool encompassing the entire length of the spike protein based on the sequence of the reference strain was used to stimulate the splenocytes (**Figure 2A**). All Omicron-based vaccine formulations, regardless of antigen dose or adjuvant, elicited an IFN-γ response above the naïve controls (*p* < 0.0001). Interestingly, the SLA-adjuvanted SmT1-O vaccine formulations induce significantly higher cellular immunity (mean of 201 and 399 spot forming cells (SFCs) with 1 and 3 µg of Ag, respectively) when compared to the AddaS03-adjuvanted formulations at equivalent antigen doses (mean of 59 and 56 SFCs, respectively; *p* < 0.001). In addition, SLA-adjuvanted vaccine containing the SmT1-O antigen induced less SFCs than the control, SLA-adjuvanted SmT1-R vaccinated mice (mean of 201 *vs*. 648 SFCs) at an equivalent antigen dose of 1 µg (*p* < 0.001). This is in contrast to our previous study where antigens based on the reference strain, Beta or Delta VOCs generated similar ELISpot responses when stimulated with a peptide pool based on the reference spike protein under the same conditions (16).

### 2.2 Humoral immunity

To elucidate the humoral immune responses induced by these vaccine formulations, SmT1-R and SmT1-O antigen-specific IgG titers in serum collected on Day 28 (**Figure 2B**) were assessed by ELISA. Overall, SLA-adjuvanted SmT1-O elicited a geometric mean titer (GMT) of 423,817 – 614,699 against SmT1-R, whereas AddaS03 formulated vaccines had a GMT range of 336,104 – 399,231, compared to the control SLA-adjuvanted SmT1-R with a GMT of 660,456. A similar range of titers were observed when assessing for IgG specific to SmT1-O, SLA-adjuvanted SmT1-O induced a GMT of 428,954 – 615,876, whereas AddaS03-adjuvanted vaccines had 289,384 – 368,311, compared to the control SLA-adjuvanted SmT1-R with a GMT of 419,242. These were all significantly higher than the baseline levels seen in the naïve control group (p<0.0001). Regardless of vaccine formulation, anti-SmT1-R and anti-SmT1-O IgG titers were largely comparable to one another with only a few statistically significant differences measured. Namely, mice receiving the SmT1-R + SLA formulated vaccine had statistically higher IgG specific to SmT1-R when compared to those receiving the AddaS03 formulated SmT1-O vaccines (1 µg SmT1-O + AddaS03, p<0.01; 3 µg SmT1-O + AddaS03, p<0.001). For the anti-SmT1-O titers, the only significant difference was seen between mice receiving 3 µg SmT1-R + SLA *vs*. 3 µg SmT1-O + AddaS03 (615,876 *vs*. 368,311; p<0.001). Given the ~97% identity of Omicron spike to the reference sequence, it is not surprising that the magnitude of responses induced by SmT1-R *vs*. SmT1-O were generally similar as the ELISA is measuring the overall polyclonal response across the length of the entire protein including the trimerization domain. As illustrated in our previous work, similar antibody titers do not necessarily equate to similar neutralization capabilities (14). As such, we next confirmed the neutralization activity of the immunized mouse sera.

### 2.3 Neutralizing activity

**Figure 3:**
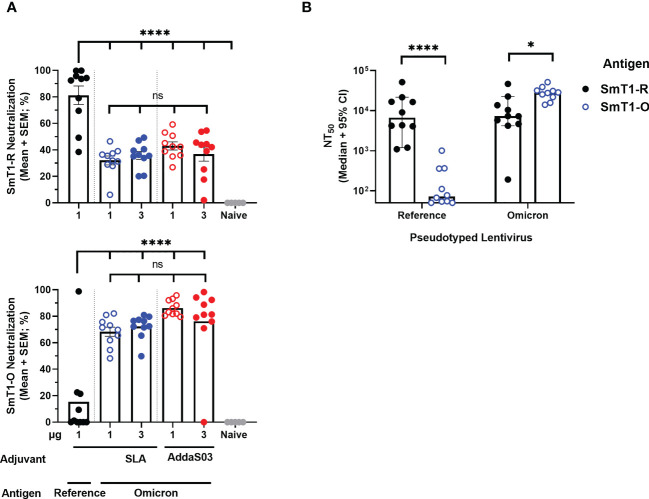
Neutralization activity towards Omicron Spike is stronger in sera of mice immunized with SmT1-O *vs*. SmT1-R-based vaccine formulations. C57BL/6 mice (n = 10/group) were immunized i.m. with SmT1-R or SmT1-O adjuvanted with SLA or AddaS03 on days 0 and 21. **(A)** Serum was collected on Day 28 and analyzed by surrogate cell-based SARS-CoV-2 spike-ACE2 binding assay at a dilution of 1 in 75 for neutralization activity against the reference strain using SmT1-R (top) and the Omicron strain using SmT1-O (bottom). **(B)** Serum from mice immunized with the SLA-adjuvanted formulations containing 1 µg SmT1-R or SmT1-O antigens were also assessed for neutralization activity against lentivirus pseudotyped with either reference (Left) or Omicron (Right) SARS-CoV-2 spike. Statistical significance of differences among groups receiving SmT1-O vaccine formulations as well as these groups *vs*. the control dose of SmT1-R are shown: ns = not significant, *p < 0.05 and ****p < 0.0001 by one-way ANOVA followed by Tukey’s multiple comparisons test **(A)** or by unpaired Student’s t-test followed by Welch’s correction **(B)**.

Initially, the Day 28 serum samples were evaluated with a surrogate cell-based SARS-CoV-2 spike-ACE2 binding assay that we had previously shown to correlate well with pseudolentivirus neutralization and SARS-CoV-2 plaque reduction assays (14, 16). Overall, the SmT1-O formulated vaccines elicited significantly higher neutralizing activity against binding of the Omicron-based spike protein to cells (68-86% mean neutralization) as compared to an average of 15% neutralization with serum of mice from the SmT1-R group (p<0.0001). The opposite trend was seen with binding of reference strain-based spike; with subpar neutralization (32-46% mean neutralization) with serum from mice immunized with formulations containing Omicron-based antigen regardless of adjuvant used, compared to 81% neutralization induced by SmT1-R+SLA (p<0.0001) (**Figure 3A**). To further confirm the reliability of the results obtained with the cell-based SARS-CoV-2 spike-ACE2 binding assay, a pseudolentiviral neutralization assay was performed with a smaller subset of samples. Specifically, the sera of mice immunized with SLA-adjuvanted formulations containing 1 µg of SmT1-R or SmT1-O antigen were tested for their ability to neutralize infection by lentiviral particles pseudotyped with either the reference or Omicron spike (**Figure 3B**). The dilution of serum required to achieve a 50% reduction in infection (neutralizing titer (NT)_50_) was calculated, with a median value of 6,681 *vs*. 72 against reference spike pseudotyped lentivirus for the SmT1-R and SmT1-O vaccinated mice, respectively (p<0.0001). On the other hand, the sera from the SmT1-R vaccinated mice had >3-fold-lower median NT_50_ against the Omicron spike pseudotyped lentivirus infection than from the mice immunized with the SmT1-O-based formulation (7,264 *vs*. 27,962; p<0.05). As with the cell-based SARS-CoV-2 spike-ACE2 binding assay, the pseudolentiviral neutralization assay confirmed that the use of a spike-based antigen from a specific variant is more capable of inducing neutralizing antibodies against that particular strain. Furthermore, despite the differences in protein sequence/characteristics with SmT1-O *vs*. the original SmT1-R (**Figure 1**), the SmT1-O antigen still proved to be highly immunogenic.

## 3 Discussion

A number of efficacious SARS-CoV-2 vaccines of different types (e.g. mRNA, adenoviral, protein subunit) have received clinical approval by regulatory authorities (19). While originally based on the sequence of the spike protein from the reference strain, bivalent mRNA vaccines containing a mixture of reference strain and Omicron or Omicron-subvariant nucleotide sequences, have been used much more recently to address emerging SARS-CoV-2 variants (20, 21). When used as a booster to reference strain-based vaccines in the clinic, these updated bivalent mRNA vaccines were initially shown to enhance neutralizing antibody titers against the circulating Omicron-based SARS-CoV-2 strains (20). Due to various concerns, including the longevity of protection and the association of adenovirus and mRNA-based vaccines with rare, but serious side effects, different vaccine platforms are still being actively evaluated to produce new vaccines to target SARS-CoV-2 variants (22). For example, a few protein subunit variant-based vaccine formulations have also been assessed clinically in the context of a booster dose (23–25). Also, bivalent or multivalent protein vaccine formulations have been used to both prime and boost immune responses within a preclinical setting, resulting in an increased breadth of the neutralization response (16, 26).

Given the global prevalence of more divergent variants, namely Omicron and its subvariants, we sought to evaluate a preclinical Omicron vaccine formulation using our novel protein production platform and SLA glycolipid adjuvant system. In addition to the underlying sequence differences, SmT1-O exhibited different protein characteristics *vs*. SmT1-R. (**Figure 1A**). To help assess antigen quality, the binding profile of SmT1-R and SmT1-O to ACE2 was analyzed (**Figure 1B**). The SmT1-O antigen demonstrated an increased affinity for ACE2, similar to that reported in the literature (17, 18). These differences may be linked to the significant structural remodelling of Omicron relative to the reference strain, whereby several variations in electrostatic interactions and new salt bridges have been reported (27). We have previously demonstrated that immunizing mice with VOC-based vaccines, namely Beta and Delta-based spike trimer antigens, provided enhanced neutralizing responses to spike proteins from the corresponding variant (16). This trend was again confirmed with our SmT1-O antigen, which as observed in two separate neutralization assays, induced stronger neutralizing responses to Omicron-based spike than a reference strain-based vaccine formulation. It is important to note that are some differences between the two neutralization assays used. First, the cell-based spike-ACE2 binding assay monitors relative abundance of SARS-CoV-2 spike binding on a trimer-by-trimer basis to the cell surface in the absence of TMPRSS2, thereby taking into account the impact of serum antibodies on receptor binding only. Meanwhile, the viral pseudoneutralization assay, which uses target cells that express both ACE2 and TMPRSS2, measures the ability of antibodies to block receptor binding, fusion and/or entry of the viral particle, as all these steps are required to mediate cell infection by pseudotyped virus and consequent expression of transgene. Remarkably, the latter assay revealed that the SmT1-O vaccine provided little measurable neutralization of the virus pseudotyped with reference spike (~93-fold less than with the SmT1-R vaccine), but a robust improvement against the Omicron spike-pseudotyped virus (~4-fold more neutralization activity than the SmT1-R vaccine). In the cell-based spike-ACE2 binding assay, the SmT1-O based formulations were not as efficient as the SmT1-R vaccine at inducing immune responses capable of blocking cellular attachment of SmT1-R protein, but there was still measurable inhibition of binding at the tested serum dilution of 1 in 75. Recent evidence indicates that unlike the parental SARS-CoV-2, the Omicron strain is not dependent on TMPRSS2 for plasma membrane fusion, rather it prefers an endocytic entry pathway (28). Thereby, differences in cell entry mechanisms could also influence the trends between reference *vs*. Omicron-pseudotyped virus above. Regardless of the differences in the assays, they both revealed the same hierarchy in neutralization between the SmT1-O *vs*. SmT1-R immunized groups against the corresponding spike proteins. It would be useful to determine the impact of Omicron-based boosters on the neutralizing responses of a population that has been imprinted with a reference-based vaccination series. The mutations and resulting conformational changes of the SmT1-O antigen may favour generation of Omicron-specific neutralizing antibodies at the expense of reference strain neutralizing antibodies (**Figure 3B**). This trend was observed within another recent evaluation of an Omicron-based mRNA vaccine, whereby the serum of vaccinated macaques exhibited a 10-20 fold higher Omicron-specific neutralization than that observed for the reference, Beta or Delta strains in a plaque reduction neutralization test (29). Interestingly, recent reports have indicated that while initial neutralizing titers against Omicron are quite robust shortly after boosting with a reference strain-based vaccine, the decline in antibody potency is more rapid for Omicron-specific neutralizing antibodies than reference strain neutralizers (30, 31). It will be important in future studies to confirm whether this bias in longevity of immune responses to different variants of SARS-CoV-2 is alleviated through the use of an Omicron-based vaccine formulation.

An ideal vaccine candidate would have a favourable safety profile, while balancing both humoral and cell-mediated immunity, both of which are involved in determining COVID-19 disease outcomes (32–35). In our study, we included a head-to-head comparison of our experimental adjuvant SLA *vs*. AddaS03, a mimetic of the AS03 adjuvant used in various influenza and SARS-CoV-2 vaccines (7). With regards to safety, while SLA has not been tested yet in humans, it has been shown in multiple preclinical studies to be a well-tolerated vaccine adjuvant in numerous animal species including mice, rats, rabbits and hamsters (12–14). As for immunogenicity, our ELISA data indicated that AddaS03 or SLA induced a comparable magnitude of SARS-CoV-2 spike specific IgG, with similar levels quantified at the 1 µg dose of antigen. However, increasing the antigen dose to 3 µg resulted in significantly higher IgG titers for SLA *vs*. AddaS03-adjuvanted formulations. We did not detect any differences in serum neutralizing activity between mice immunized with SLA or AddaS03-adjuvanted formulations. In agreement with a previous report (16), we did see that SLA-adjuvanted vaccines with SmT1-O induced significantly more spike-specific cellular responses than similar AddaS03-adjuvanted formulations. However, these were lower than seen with an equivalent SmT1-R-based formulation (p<0.001). This may signify the presence of mutations in SmT1-O that alter the ability of potentially immunogenic peptides to be presented by MHC class I haplotypes present in C57BL/6 mice and/or the ability of the reference-based peptide pool used above for the ELISpot to activate T cells recognizing Omicron spike-specific epitopes. The use of an Omicron-specific peptide pool could indicate whether T cell responses were redirected to other epitopes, but this was beyond the scope of this study and may not be directly relevant to the clinical situation due to the altered specificity of mouse *vs*. human MHC molecules.

While the first approved SARS-CoV-2 vaccines were based on the reference strain spike sequence, originally identified in Wuhan, China, there are currently two clinically available bivalent mRNA vaccines (50% reference strain and 50% variant composition) that were initially shown to generate slightly improved neutralizing activity against Omicron or subvariants thereof (20, 21). However, recent reports by independent researchers have not confirmed these observations, indicating that there was no significant benefit in neutralizing titer against Omicron variants after boosting with bivalent *vs* reference strain mRNA vaccines (36–38). The potential benefit from these bivalent vaccines over traditional reference strain boosters may be limited by original antigenic imprinting (36–38). It remains to be determined if using monovalent variant vaccines lacking reference-based antigens could better focus the response to the targeted variant, instead of just boosting responses generated previously to the original antigen. Further investigation may be necessary to evaluate the longevity of these neutralizing responses towards variants, as this may be where variant-specific formulations demonstrate an advantage over reference strain-based vaccines (30, 31).

Waning immunity and the continued emergence of new immune evasive variants necessitates the development of updated vaccines better capable of protecting against the circulating strains of SARS-CoV-2. Here, as an example of the role of updated vaccines, we have validated the potential of Omicron-based spike protein subunit vaccines. Overall, a more balanced cell-mediated and humoral neutralizing response was evoked by SLA-adjuvanted vaccines, whereas AddaS03-containing formulations generated similar antigen-specific humoral but weaker cellular immune responses. Updating the antigen sequence did not strongly impact the overall antigen-specific IgG titers, rather the quality of the neutralizing responses. This clearly illustrates the potential to refine antigen-specific immunological responses through the use of different antigens and/or adjuvants in vaccine formulations. In future studies, the cross-neutralizing capacity of these vaccines to Omicron subvariants could be interesting to assess, though with the rapidly changing profile of circulating strains, its relevance is not entirely clear. It will also be important to attempt to mimic the current scenario in the clinical population by evaluating the above SmT1-O-based vaccine formulations as a booster regimen in subjects that have already received a reference strain-based vaccination series.

## 4 Materials and methods

### 4.1 Antigens

SmT1-R and SmT1-O constructs are SARS-CoV-2 spike trimers produced in CHO^55E1^ cells as described previously (15). Briefly, the SARS-CoV-2 reference strain spike ectodomain sequence (amino acids 1-1208 derived from Genbank accession number MN908947) was codon-optimized for Chinese Hamster Ovary (CHO) cells and synthesized by GenScript. Within the construct, the spike glycoprotein was preceded by its natural N-terminal signal peptide and fused at the C-terminus to human resistin (accession number NP_001180303.1, amino acids 23-108) and purification tags (FLAG-(His)_6_ for SmT1-R and dual-Strep-(His)_6_ for SmT1-O). Mutations were added to stabilize the generated spike protein as previously described; amino acids 682-685 (RRAR) and 986-987 (KV) were replaced with GGAS and PP, respectively (39, 40). Constructs were cloned into the pTT5^®^ plasmid. The expression construct for Omicron spike variant was prepared by re-synthesizing and replacing restriction fragments containing associated mutations (A67V, 69-70del, T95I, GVYY142-145D, NL211-212I, ins214EPE, G339D, S371L, S373P, S375F, K417N, N440K, G446S, S477N, T478K, E484A, Q493R, G496S, Q498R, N501Y, Y505H, T547K, D614G, H655Y, N679K, P681H, N764K, D796Y, N856K, Q954H, N969K, L981F). Supernatants of transiently transfected CHO^55E1^ cells were harvested at 7 days post-transfection as described (15).

For SmT1-O purification, cleared supernatant was first purified by IMAC using Nickel Sepharose Excel resin (GE Healthcare), as described (15). IMAC eluate was subjected to a secondary purification step using a 5-ml StrepTrap HP column (GE Healtchare). Approximately 12 mg of IMAC eluate was loaded on the StrepTrap column, equilibrated in DPBS adjusted to pH 7.8, at a flow rate of 2-3 ml/min. Following a washing step (4 column volumes (CV) of DPBS pH7.8), spike protein was eluted with DPBS + 2.5 mM desthiobiotin, pH 7.8. Buffer was exchanged for DPBS, pH 7.8, and protein was concentrated to ~1 mg/ml using Amicon Ultra 15 (50 kDa cutoff) centrifugal filters (EMD Millipore).

Recombinant spike protein was purified using AVIPure-COV2S resins (Avitide, Lebanon, New Hampshire) according to the manufacturer’s instructions. Briefly, 0.2 μm-filtered supernatant was loaded on a column equilibrated with DPBS. The column was washed once with 10 CV of DPBS and protein eluted with 50 mM Bis-Tris, 1 M Arginine-HCl, pH 6.0. The fractions containing eluted proteins were pooled and buffer exchanged for DPBS using desalting columns (GE Healthcare). Purified protein was quantified by spectrophotometry (A280) using extinction coefficient calculated based on the amino acid composition. Peptide mass spectrometry was used to confirm the identity of the expected variant protein in SmT1-R and SmT1-O samples.

Purified proteins were analyzed by SDS-PAGE using NuPAGE 4-12% Bis-Tris gels (Invitrogen) followed by Coomassie Blue staining or western blotting. Primary antibodies for western blotting were from Cell Signaling (E7M5X) or produced in-house (V_H_H S2A4) (41). The absence of endotoxin contamination was verified using Endosafe cartridge-based Limulus amebocyte lysate tests (Charles River Laboratories, Charleston, SC, USA).

### 4.2 Surface plasmon resonance binding assays

Binding affinity (K_D_) of purified monomeric ACE2 to the trimerized S-proteins of SARS-CoV-2 was determined using a Biacore T200 surface plasmon resonance (SPR) instrument (Cytiva, Marlborough, MA). SPR experiments were carried out at 25°C using PBS containing 0.05% Tween 20 (Teknova, Hollister, CA) with added 3.4 mM ethylenediaminetetraacetic acid (EDTA) and 0.05% Tween 20 as running buffer (PBST). Production and purification of recombinant human ACE2 protein was carried out as described (42). The binding analysis was carried out in two steps, first using indirect capture of the S-protein trimer *via* the human resistin trimerization domain immobilized onto the SPR surface, followed by flowing the ACE2 over top to generate the binding sensorgrams. The capture surface was generated using an anti-resistin single-domain (VHH) antibody fused to a human IgG1 Fc (41). This antibody was diluted to 10 µg/mL in 10 mM sodium acetate immobilization buffer pH 4.5 (Cytiva, Marlborough, MA) and immobilized to approximately 2000 RUs using the Immobilization Wizard for NHS/EDC amine coupling within the Biacore T200 Control instrument software (v2.0.1). The S-protein trimers under analysis were diluted to 10 µg/mL in PBST and captured onto the anti-resistin surface at 10 µL/min for 180 s. The ACE2 – S-protein interactions were then assessed using single cycle kinetics analysis with three concentrations, using a 5-fold dilution from the top concentration of 200 nM. The ACE2 was injected at 50 µL/min over captured spike protein with a contact time of 150 s at each concentration and a 600 s dissociation. At the end of dissociation, the anti-resistin surfaces were regenerated with a 30 s pulse of 10 mM glycine pH 1.5 at 30 uL/min. Sensorgrams were double referenced to the blank anti-resistin sensor surface and analyzed for binding affinity and kinetics using the 1:1 binding model in the Biacore T200 Evaluation software (v3.0.2).

### 4.3 Immunization and sample collection

Female C57BL/6 mice (6–8 weeks old) were obtained from Charles River Laboratories (Saint-Constant, Canada). Animals were maintained at the small animal facility of the NRC Canada in accordance with the guidelines of the Canadian Council on Animal Care.

Mouse experiments were conducted with n = 10 per group. Antigen and adjuvant vaccine components were admixed and diluted in PBS (Thermo Fisher Scientific, Waltham, MA, USA) prior to administration in a final volume of 50 µL per dose. SLA archaeosomes are proprietary NRC adjuvants that were prepared as previously described (43). Levels of endotoxin in the SLA archaeosomes were verified by the Endosafe cartridge-based Limulus amebocyte lysate test (Charles River Laboratories) and confirmed to be <0.1 EU per mg. AddaS03 (Invivogen, San Diego, CA, USA) was prepared as per the manufacturer’s instructions.

Animals were immunized by intramuscular (i.m.) injection (50 µL) into the left tibialis anterior muscle on days 0 and 21 with various vaccine formulations as described above. On day 28, mice were anesthetized with isoflurane and then euthanized by cervical dislocation prior to collection of spleens for measurement of cellular immune responses by IFN-γ ELISpot. Mice were bled *via* the submandibular vein on day 28 with recovered serum used for quantification of antigen-specific IgG antibody levels and neutralization assays. Samples were simultaneously collected from 10 naïve animals for the assessment of background immune responses. Each of the samples from the individual mice was tested separately in the various readouts.

### 4.4 Anti-spike IgG ELISA

Anti-spike total IgG titers in serum were measured by indirect ELISA with SmT1-R or SmT1-O as previously described (14, 44). Briefly, 96–well high-binding ELISA plates (Thermo Fisher Scientific) were coated with 0.3 µg/mL SmT1 protein diluted in PBS. Serum samples were serially diluted 3.162-fold and added to the plates to allow for binding of antibodies to the protein. Bound IgG was detected with goat anti-mouse IgG -HRP (1:4000, Southern Biotech, Birmingham, AL, USA) prior to the addition of the substrate o-phenylenediamine dihydrochloride (Sigma-Aldrich). Bound IgG Abs were detected spectrophotometrically at 450 nm. Titers for IgG in serum were defined as the dilution that resulted in an absorbance value (OD450) of 0.2 and was calculated using XLfit software (ID Business Solutions, Guildford, UK). Samples that did not reach the target OD were assigned the value of the lowest tested dilution (i.e., 100) for analysis purposes. No detectable titers were measured in serum samples from naïve control animals.

### 4.5 IFN- γ ELISpot

IFN- γ ELISpot was also conducted as previously described (14, 45). The levels of spike glycoprotein specific T cells were quantified by ELISpot using a mouse IFN-γ kit (Mabtech Inc., Cincinnati, OH, USA). A spike peptide library (JPT Peptide Technologies GmbH) based on the reference strain sequence and consisting of 315 peptides (15mers overlapping by 11 amino acids with last peptide consisting of a 17mer) was used to stimulate splenocytes isolated from each of the mice. The library was split into three subpools and used to separately stimulate 4 × 10^5^ cells in duplicate at a final concentration of 2 µg/mL per peptide. Cells were also incubated without any stimulants to measure background responses. Spots were counted using an automated ELISpot plate reader (Cellular Technology LTD, Beachwood, OH, USA). For each animal, values obtained with media alone were subtracted from those obtained with each of the spike peptide pools, and then combined to yield an overall number of antigen-specific IFN-γ+ SFC/10^6^ splenocytes per animal.

### 4.6 Cell-based SARS-CoV-2 spike-ACE2 binding assay

The ability serum to neutralize the binding of labeled SARS-CoV-2 spike trimers (SmT1) ACE2-expressing cells was performed similarly as previously described (14). The main difference herein is the use of the HEK-293T-hACE2 cells (BEI Resources NR-52511) instead of Vero E6 cells. Indicated dilutions of mouse serum were mixed with 250 ng of biotinylated spike and 1 × 10^5^ HEK-293T-hACE2 cells. The amount of bound spike was quantified using Streptavidin-phycoerythrin conjugate analyzed on an LSR Fortessa (Becton Dickinson). For illustration purposes, samples with calculated values ≤0 were assigned a value of 0.

### 4.7 Pseudovirus neutralization assay

Pseudovirus neutralization assay was performed in 384-well plate format adapted from previously described protocol and modification (46, 47). Briefly, 4-fold serial dilutions of the serum samples were incubated with diluted virus at a 1:1 ratio for 1 h at 37 °C before addition to HEK293-ACE2/TMPRSS2 cells obtained from BEI Resources repository of ATCC and the NIH (NR-55293). Infectivity was then measured by luminescence readout per well. Bright-Glo luciferase reagent (Promega, E2620) was added to wells for 2 min before reading with a PerkinElmer Envision instrument. Neutralization Titer 50 (NT50) were calculated with nonlinear regression (log[inhibitor] versus normalized response – variable slope) with the 100% and 0% constraint. Pseudotyped lentiviral particles were produced expressing the SARS-CoV-2 variant spikes under CMV promotor and were packaged onto lentiviral vectors obtained through BEI Resources, NIAID, NIH: SARS-Related Coronavirus 2, Wuhan-Hu-1 (GenBank # NC_045512) Spike-Pseudotyped Lentiviral Kit, NR-52948. pTwist-SARS-CoV-2 Δ18 B.1.1.529 expressing SARS-CoV-2 omicron variant was a gift from Alejandro Balazs (Addgene plasmid # 179907).

### 4.8 Statistical analysis

Data were analyzed using GraphPad Prism version 8 (GraphPad Software). Statistical significance of the difference between groups was calculated by one-way analysis of variance followed by *post hoc* analysis using Tukey’s (comparison across all groups) multiple comparison test or unpaired t test with Welch’s correction where indicated. Data were log-transformed (except for data from surrogate cell-based spike-ACE2 binding assay) prior to statistical analysis. For all analyses, differences were considered to be not significant with p > 0.05. Significance was indicated in the graphs as follows: *p < 0.05, **p < 0.01, ***p < 0.001, and ****p < 0.0001.

## Data availability statement

The original contributions presented in the study are included in the article/supplementary materials, further inquiries can be directed to the corresponding author/s.

## Ethics statement

The animal study was reviewed and approved by Institutional Review Board (NRC Human Health Therapeutics Animal Care Committee).

## Author contributions

BA, TR, MS, YD and MM conceived and designed the studies. TR, MS, NR, YC-D, LD, RD, BH, JB, IK, JH, UH, SR, AL and YD contributed to the synthesis/validation of the vaccine components and/or execution of the experiments. BA, TR and MS analyzed the data. TR, BA and MM took the lead in writing the manuscript. All authors contributed to the article and approved the submitted version.
